# Development of a *Pseudomonas aeruginosa* Agmatine Biosensor

**DOI:** 10.3390/bios4040387

**Published:** 2014-10-29

**Authors:** Adam Gilbertsen, Bryan Williams

**Affiliations:** Division of Pulmonary, Allergy, Critical Care and Sleep Medicine, University of Minnesota, 420 Delaware St. SE MMC 276, Minneapolis, MN 55455, USA; E-Mail: gilbe398@umn.edu

**Keywords:** agmatine, *Pseudomonas*, arginine decarboxylase, bioluminescence, polyamines

## Abstract

Agmatine, decarboxylated arginine, is an important intermediary in polyamine production for many prokaryotes, but serves higher functions in eukaryotes such as nitric oxide inhibition and roles in neurotransmission. *Pseudomonas aeruginosa* relies on the arginine decarboxylase and agmatine deiminase pathways to convert arginine into putrescine. One of the two known agmatine deiminase operons, *aguBA*, contains an agmatine sensitive TetR promoter controlled by AguR. We have discovered that this promoter element can produce a titratable induction of its gene products in response to agmatine, and utilized this discovery to make a luminescent agmatine biosensor in *P. aeruginosa*. The genome of the *P. aeruginosa* lab strain UCBPP-PA14 was altered to remove both its ability to synthesize or destroy agmatine, and insertion of the luminescent reporter construct allows it to produce light in proportion to the amount of exogenous agmatine applied from ~100 nM to 1mM. Furthermore it does not respond to related compounds including arginine or putrescine. To demonstrate potential applications the biosensor was used to detect agmatine in spent supernatants, to monitor the development of arginine decarboxylase over time, and to detect agmatine in the spinal cords of live mice.

## 1. Introduction

**Figure 1:**
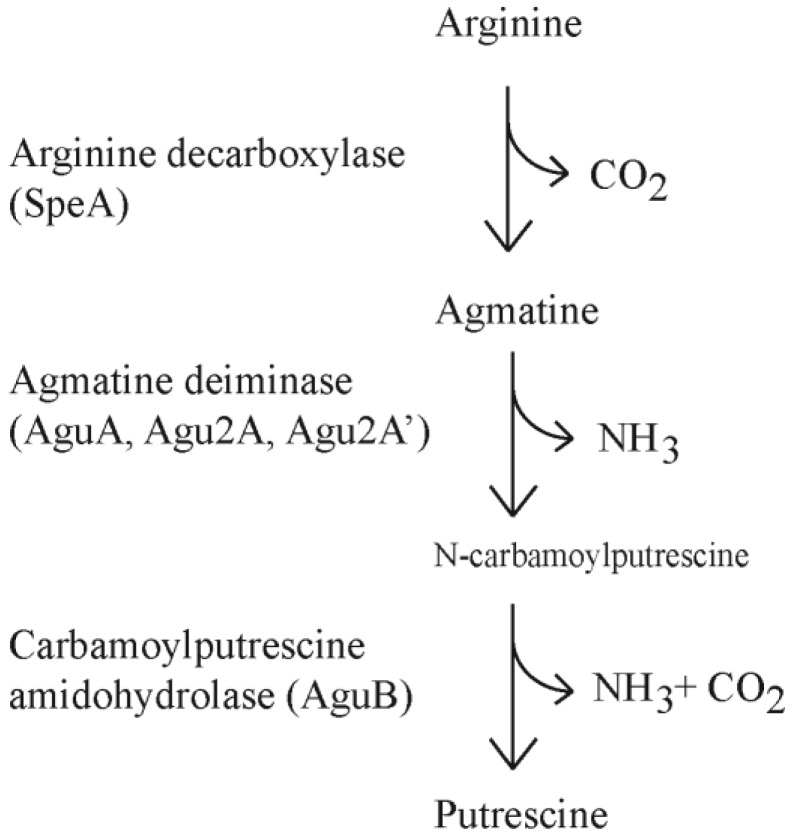
The arginine decarboxylase and agmatine deiminase pathways.

The polyamines (putrescine, cadaverine, spermine, spermidine) are found throughout all five kingdoms of living organisms and are attributed to diverse and frequently vital cellular processes. In most forms of life polyamine production is critical for cellular division and DNA synthesis. Polyamines are thought to stabilize DNA and RNA to allow for more efficient replication and transcription although the exact mechanism remains elusive [1,2]. Generation of polyamines occurs through conserved pathways including the ornithine decarboxylase pathway (ODC) and arginine decarboxylase pathway (ADC). In the ODC pathway, ornithine is converted to putrescine whereas the ADC pathway converts arginine to agmatine. Agmatine can be converted to putrescine in one step through agmatinase or two steps through agmatine deiminase (AgDi) and N-carbamoylputrescine amidohydrolase (Figure 1).

Agmatine is considered a “pre-polyamine” and has been shown to have a number of diverse roles in higher order eukaryotes. Agmatine functions as a neurotransmitter through interaction with α2-adrenergic, nicotonic, serotonin and imidazoline-receptors in the central nervous system, and has been putatively shown to selectively block NMDA receptor channels [3,4,5]. Agmatine has been shown to act as a neuroprotective molecule by inhibiting the NOS-2 protein, and consequently nitric oxide (NO), in macrophages and astroglial cells [6,7]. It is a vasoactive mediator in endothelium, cytoprotective to cells undergoing oxidative stress, and neuroprotective in models of stroke and epilepsy [7]. A vast arena of potential therapeutic applications is being explored given these mechanisms of action [3].

Agmatine is found in plants as well and the generation of polyamines may rely more on the ADC pathway than in animals as many plants have reduced or absent ODC [8]. Little is known about agmatine in plant physiology other than its precursor role as a polyamine. Agmatine expression was recently implicated as a mechanism of resistance to clubroot in *Arabidopsis* [9].

Polyamine metabolism is thoroughly described in the bacterial literature. ADC and ODC pathways are prevalent in most species described as most bacteria rely heavily on putrescine for cellular division [1]. The positively charged polyamines, putrescine and spermidine, have been observed to counter the effects of antibiotic and oxidative stressors by binding to the lipopolysaccharide (LPS) of *Pseudomonas aeruginosa*. This restores membrane stability in negatively charged (*i.e*., Mg^2+^ limited, DNA rich, *etc*.) environments and potentially suggests a relationship of polyamines with antibiotic resistance and the formation of biofilms [10,11]. Agmatine is not implicated in many cellular processes in bacteria outside of metabolism, although our laboratory recently implicated exogenous agmatine in the enhancement of biofilm formation in *P. aeruginosa* [12]. An agmatine-induced transcriptosome has been studied in *P. aeruginosa* and most of the implied functions are metabolic and related to metabolism of related compounds and not higher order cellular functions [13].

In *P. aeruginosa* agmatine can be utilized as the sole carbon source via the ADC pathway when metabolized with the gene products of the *aguBA* operon: agmatine deiminase (coded by *aguA*) and N-carbamoylputrescine amidohydrolase (coded by *aguB*) [14]. Our lab discovered an alternate operon for agmatine metabolism (*agu2ABCA’*) that appears to have little impact on agmatine levels compared to *aguBA* [12]. In this previously published work we also demonstrate the *aguBA* operon appears to be universally present in *P. aeruginosa* isolates whereas the *agu2ABCA’* isolate was present in ~20% of isolates when screened by PCR. The *aguBA* operon is regulated by a transcription suppressing protein, AguR, that binds the promoter between the -35 and -10 sites [15]. The product of *aguR* belongs to the TetR family of transcriptional regulators and remains bound to the *aguBA* promoter site, inhibiting transcription until agmatine binds to the protein, releasing it from the promoter. Disruption of the *aguR* gene results in constitutive expression of the *aguBA* operon.

In this work we fused the genetic elements from the *aguBA* operon responsible for agmatine detection to the bioluminescent reporter mini-CTX-lux construct and inserted this into a *P. aeruginosa* mutant incapable of agmatine synthesis or metabolism. This allows for agmatine detection and reporting through bioluminescence without manipulating the environmental agmatine levels. This creates a relatively easier and more cost efficient way to monitor and quantify agmatine levels compared to mass spectrometry, capillary electrophoresis, and chemical chromogens, with the added potential for *in vivo* applications. As *P. aeruginosa* is ubiquitous and shows pathogenicity towards multiple hosts, it is ideally suited for *in vivo* measurement in a number of models.

## 2. Experimental Section

### 2.1. Bacterial Strains and Plasmids

*P. aeruginosa* strain PA14 [16], its agmatine mutants (see Table 1), and the *P. aeruginosa* clinical isolates were either grown in Luria Bertani media or RPMI media as indicated in the text. All growth occurs at 37 °C with orbital shaking for liquid cultures at 225 rpm. For UPLC-MS/MS analysis of spent supernatant RPMI was used, as it is a defined medium without added agmatine and did not suffer the same analyte suppression as LB.

Construction of most of the PA14 agmatine mutants was described previously [12]. Table 2 describes the plasmids made in this work. The removal of the *speA* gene from the chromosome occurred using the pEX18 system as previously described [17]. *SpeA* was amplified from PA14 genomic DNA and inserted into pEX180-Ap utilizing the native *EcoR*I and *Hind*III sites within the fragment. To create the *speA* knockout the plasmid was digested with *Sph*I and *EcoR*V, and blunted with the NEB “Quick blunting” enzyme kit. This removed 744 bp near the center of *speA* leaving flanking regions of 1035 bp and 842 bp on either side. The final construct was transformed into the mating *E. coli* strain SM10 and subsequently mated with PA14 rendering the desired SpeA knockout phenotype. The genomic DNA of the resulting mutants was screened via PCR. PCR reactions were performed with the GC-Rich PCR system (Roche) and using forward primer 5ʹ-TTGTTGACCTGGCCCGTCGA-3ʹ and reverse primer 5ʹ-GGGAAGCGGAAATGAAGGGG-3ʹ to both generate and screen clones and mutants.

To generate the arginine decarboxylase expression strain the gene for *speA* was amplified from PA14 genomic DNA using forward primer 5ʹ-CACCATGGCCGCTCGACGGACT-3ʹ and reverse primer 5ʹ-GGACAGGTACGCCGAGCGG-3ʹ and then cloned into the pBAD Directional TOPO vector as described by the manufacturer (Life technologies, Green Island, NY, USA). The SpeA gene product was expressed and purified per manufacturer instructions.

**Table 1:** Bacterial strains.

Parent strain	Phenotype	Genotype	Source or reference
PA14	*Wild-type*	Wild-type	[18]
	WT *Agmatine Reporter*	*aguRB*-CTX-Lux	This work
	WT *Reporter Vector Control*	CTX-Lux	This work
	AgDi Knockout	*aguA:gm*, *∆agu2ABCA’*	[12]
	AgDi Knockout, *Agmatine Reporter*	*aguA:gm*, *∆agu2ABCA’*, *AguRB*-CTX-Lux	This work
	AgDi Knockout, *Reporter Vector Control*	*aguA:gm, ∆agu2ABCA’*, CTX-Lux	This work
	Arginine Decarboxylase Knockout	*∆speA*	This work
	Arginine Decarboxylase Knockout, *Agmatine Reporter*	∆*speA, AguRB*-CTX-Lux	This work
	Arginine Decarboxylase Knockout, *Reporter Vector Control*	*∆speA*, CTX-Lux	This work
	AgDi Knockout, Arginine Decarboxylase Knockout	*aguA:gm*, *∆agu2ABCA’*, ∆*speA*	This work
	**Agmatine Biosensor**-AgDi Knockout, Arginine Decarboxylase Knockout, *Agmatine Reporter*	*aguA:gm*, *∆agu2ABCA’*, *∆speA*, AguR-CTX-Lux	This work
	AgDi Knockout, Arginine Decarboxylase Knockout, *Reporter Vector Control*	*aguA:gm*, *∆agu2ABCA’*, *∆speA*, CTX-Lux	This work
*E. coli* Top10	Competent cells for cloning purposes	*F- mcrA Δ(mrr-hsdRMS-mcrBC) φ80lacZΔM15 ΔlacX74 nupG recA1 araD139 Δ(ara-leu)7697 galE15 galK16 rpsL(Str^R^) endA1 λ^-^*	Invitrogen
*E. coli* SM10	Vehicle for conjugative mating to *P. aeruginosa*	*KmR*, *thi-1*, *thr*, *leu*, *tonA*, *lacY*, *supE*, *recA::*RP4-2-Tc*::Mu*, *pir*.	[19]

**Table 2:** Plasmids.

Plasmid	Description	Features	Source or reference
Mini-ctx-lux	*luxCDABE* based reporter vector with site specific integration at *attB* site in *P. aeruginosa* chromosome	See Reference	[20]
pEX18-Ap	Gene replacement vector	See Reference	[17]
CTXnoT7	Contains Lux operon, T7 promoter removed to reduce background expression	No T7	This work
*AguRB*-lux	Contains Lux operon induced by agmatine via the inserted promotion system of the primary agmatine deiminase, T7 promoter removed to reduce background expression	No T7, *AguRB* promoter fusion to *luxCDABE*	This work
pBAD202	Arabinose induced expression vector	See manufacturer	Invitrogen
SpeBAD	*speA* from PA14 cloned into MCS of pBAD202	Arabinose induced expression vector of SpeA	This work
speA KO	pEX18-Ap based cloning vector with *speA* knockout construct	Designed for disruption of chromosomal *speA* in *P. aeruginosa*	This work

The luminescent reporter plasmid (*aguRB:lux*) was created by inserting the *aguBA* transcriptional element into the mini-ctx-lux vector as previously described [20]. The T7 promoter upstream of the multiple cloning sites in mini-ctx-lux was removed by site directed mutagenesis (Mutagenex, Piscataway, NJ, USA) to reduce background luminescence. The *aguR-B* fragment was amplified from PA14 genomic DNA using forward primer 5ʹ-GCAAGCTTTGGCGTCCAATAGCCGCTCAC-3ʹ and reverse primer 5ʹ-GCGAATTCAGTTCCTGGATCAGGATGATCTGC-3ʹ. The forward primer includes a *Hind*III site and the reverse primer includes a *Eco*R1 site which were used to clone the PCR fragment into the mini-ctx-lux vector.

### 2.2. Development of the Agmatine Biosensor

The luminescent reporter construct plasmid (*aguRB:lux*) was inserted into the genome of a PA14 mutant devoid of all genes for agmatine metabolism (Δ*speA, aguA:gm, Δagu2ABCA’*) as described in the manuscript concerning ctx-lux [20]*.* All analyses of the agmatine reporter construct were compared to identical mutants with the mini-ctx-lux vector alone to establish background luminescence. See the results section for more details on the construction of this biosensor.

### 2.3. Mass Spectrometry Measurement of Agmatine

The measurement of agmatine by mass spectrometry has been described by our lab in a previous publication [12], however we have made substantial modifications to the technique that will be described in another manuscript currently submitted. The following briefly describes the major differences in our technique:

A Waters Acquity UPLC/triple quadrupole mass spectrometer (Waters, Milford, MA, USA) was used for determination of agmatine. For standardization, eight levels of calibration mixtures ranging from 0 ng/mL to 10,000 ng/mL were prepared for agmatine and isotopic agmatine (^13^C_5_,^15^N_4_-agmatine) was used as an internal standard. Isotopic agmatine was created by reacting isotopic arginine with purified arginine decarboxylase from *P. aeruginosa* which was purified from the *speA* expression strain described above. These solutions were then analyzed by UPLC-MS/MS, and the data were subjected to a linear least squares analysis. The peak area ratios of analyte:internal standard measured in samples (prepared as described below) spiked with a fixed relative amount of internal standard equal to that present in the standard solutions were then used in conjunction with the calibration curves to determine the concentration of agmatine in the samples. Limits of detection (LOD) and quantitation (LOQ) were calculated by determining the signal-to-noise values for samples spiked with 50 ng/mL agmatine and extrapolating to the concentration at which the signal-to-noise value was 10 for LOQ or 3 for LOD.

Sample preparation was as follows: 100 µL of sample spiked with isotopic agmatine was mixed with 200 µL of ice-cold isopropanol and chilled to −20 °C for 5–8 h. The sample was centrifuged at 21,000 × *g* and the supernatant separated from the proteinacious pellet to a Amicon Ultra 3 kDa MW cutoff filtration column (Millipore). This column was centrifuged at 14,000 × *g* for 4–6 h to recover at least 100 mL of filtrate. To 100 µL of filtrate, 15 µL of borate buffer (pH 9.5) was added, followed by 15 µL of 10 mM 4-Fluoro-7-nitrobenzofurazan (NBD-F, Sigma) in acetonitrile. The sample was mixed and placed at 60 °C for 10 min. After incubation the sample was placed on ice, then treated with 20 mL of 0.3% formic acid within 2 min to stabilize the NBD-derivatized analytes. The sample was centrifuged for 5 min at 21,000 × *g*, and the supernatant centrifuged through an Ultrafree-MC GV filter column (Millipore) for final particulate removal prior to analysis by UPLC-MS/MS.

### 2.4. Bioassay Technique

A 96 well plate based bioassay was created to rapidly and qualitatively assess agmatine concentration in a variety of contexts. After overnight incubation the agmatine biosensor was diluted to an OD_600_ of 1.0, approximately equal to 1 × 10^9^ CFU/mL, and then further diluted to a final concentration of 1 × 10^6^ total organisms per well in a total volume of 100 µL on a white, clear bottom 96 well plate (BRANDplates). An additional 100 µL of analyte is added to the well before incubation at 37 °C incubator for 3 h. The plate is then placed in the preheated plate reader, shaken for 3 s and read for OD_600_ and luminescence. Plate based luminescence was measured in a SpectraMax M3 spectrophotometer (Molecular Devices, Sunnyvale, CA, USA). Luminescence is reported as relative units (RLU) and is divided by the absorbance reading to give a ratio of RLU to the concentration of bacteria.

The co-culture experiment was setup to include equal concentrations (1 × 10^6^ CFU) and volumes (100 μL) of the *P. aeruginosa* agmatine biosensor and the *E. coli* expression strain harboring inducible *speA*. Arginine was added to a final concentration of 1 mM to serve as a substrate for induced arginine decarboxylase. These were added into a 96 well plate and incubated for 10 h to allow for expression of arginine decarboxylase. The luminescence was measured as described above.

### 2.5. Spinal Cord Infection

Male ICR-CD1 were anesthetized with a ketamine (75 mg/kg), xylazine (5 mg/kg), and acepromazine (1 mg/kg) mixture. The mice were shaved and inoculated with the agmatine biosensor (1E7 total organisms, assessed by OD_600_ and confirmed by colony count) and simultaneously combined with agmatine in 0.9% saline (1 mM to 1 µM final concentrations) by direct intrathecal lumbar puncture as described for mice with a total volume of 10 µL [21,22]. After inoculation the mice were monitored for responsiveness and re-anesthetized as necessary. The mice were imaged for luminescent signal at 1 h intervals for 3 h in a Xenogen Spectrum imager using Living Image 4.4. Exposure time for all images was 3 min at medium binning. To ensure the effects of the anesthesia, isoflurane (3%, 3 L/min) was administered through nosecones within the imager during each image capture.

## 3. Results and Discussion

### 3.1. Construction of a Bioluminescent Agmatine Reporter Construct for Use in P. aeruginosa

**Figure 2:**
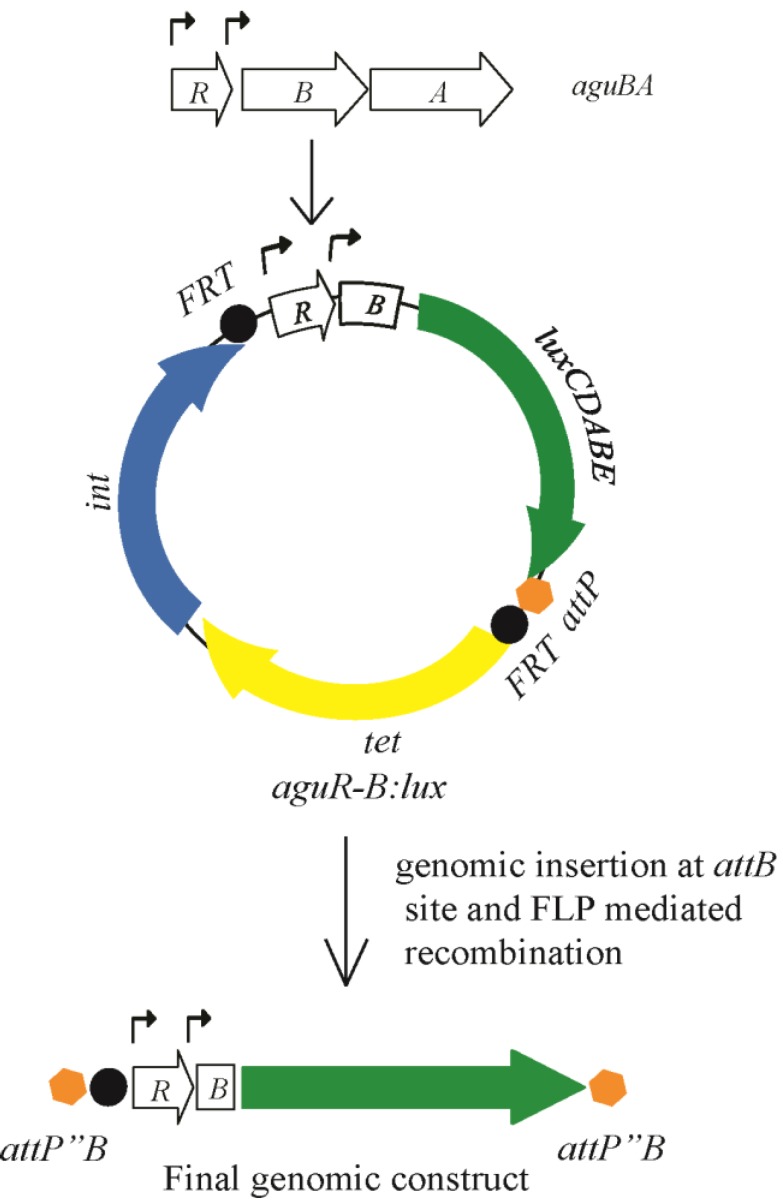
Construction of the *aguRB:lux* reporter plasmid for integration into the *P. aeruginosa* chromosome. See text for details.

The *aguBA* operon in *P. aeruginosa* is activated in the presence of agmatine. In our work with this operon we determined it to be inducible in liquid culture at any phase of growth, although preferentially during rapid, logarithmic phase growth. Our initial report of this activity was done using a beta-galactosidase reporter (min-ctx-*lacZ*) designed to harbor a promoter element and integrate into the *P. aeruginosa* chromosome at a universally present *attB* site that does not disrupt any coding open reading frames. An alternate version of this construct (mini-ctx-*lux*) was created to induce luminescence instead of beta-galactosidase activity, allowing for real time detection of transcription with a luminometer. We modified the mini-ctx-*lux* plasmid further to remove a residual T7 promoter upstream of the multiple cloning sites that created a significant background luminescence. We then cloned the PCR fragment containing the entire *aguR* sequence and the beginning portion of *aguB* to create a transcriptional fusion with the *luxCDABE* operon as shown in Figure 2. This plasmid, and its empty vector control version, was then cloned into a number of *P. aeruginosa* agmatine mutants to determine the presence of agmatine within the cell.

### 3.2. Behavior of Biosensor Construct in Mutants of the Arginine Decarboxylase and Agmatine Deiminase Pathways

**Figure 3:**
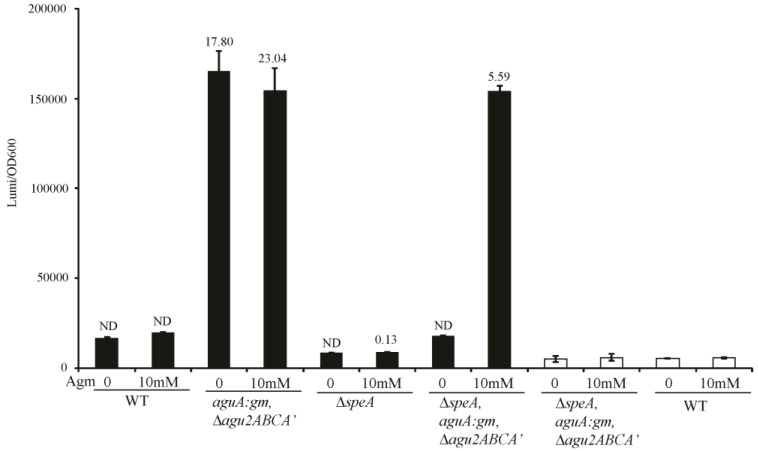
Agmatine response element behavior in mutants of the arginine decarboxylase and agmatine deiminase pathways. The agmatine “reporter construct” was inserted into the genomes of select mutant combinations in *P. aeruginosa* known to contribute to agmatine metabolism. Where indicated these mutant/reporter combinations were grown with or without agmatine. Filled boxes represent strains containing the *aguRB-lux* agmatine response element in their genomes, unfilled boxes contain the empty lux operon vector integrated into their genome. The y-axis represents relative luminescence/OD_600_ of each well at 3 h of growth. Values above the filled bars are agmatine concentrations as determined by UPLC-MS/MS in micromolar. Error bars represent SEM of three wells. ND—none detected by mass spectrometry.

In our work of the arginine decarboxylase and agmatine deiminase systems of *P. aeruginosa,* we have created mutants of every gene shown to play a role in agmatine metabolism. We have generated combinations of these mutations to create strains with divergent agmatine metabolic fates. The *aguA:gm*, Δ*agu2ABCA’* mutant should not be able to metabolize its own synthesized agmatine, or any agmatine supplemented to it. The Δ*speA* mutant should not contain any intrinsic agmatine, but should still metabolize supplemented to it. Finally the Δ*speA*, *aguA:gm*, Δ*agu2ABCA’* triple combination mutant should not be able to synthesize agmatine, or metabolize supplemented agmatine. Integration of the *aguRB:lux* reporter plasmid into these mutants confirms the expected phenotype from each mutation (Figure 3). The assays include luminescence normalization to OD_600_ to account for differences in cell number per well, which would change the luminescence intensity. We validated the presence of agmatine in these supernatants using UPLC-MS/MS, which demonstrates a grossly accurate depiction of the data trends observed with luminescence.

### 3.3. Validation of the ΔspeA, aguA:gm, Δagu2ABCA’, aguRB:lux Mutant as an Agmatine Biosensor

**Figure 4:**
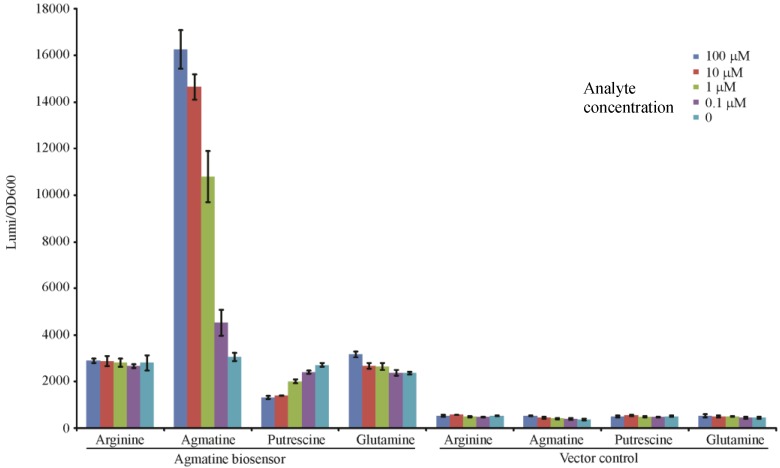
Agmatine titration in the agmatine biosensor. Each well contains 1 × 10^6^ CFU of either the agmatine biosensor (Δ*speA*, *aguA:gm*, Δ*agu2ABCA’, aguRB:lux*) or its vector control (Δ*speA*, *aguA:gm*, Δ*agu2ABCA’, ctx:lux*) in 200 μL of LB broth with the indicated concentration of stimulant. The y-axis shows the relative luminescence/OD_600_ at 3 h of growth. Error bars are SEM of three wells.

In our analysis we determined that the mutant containing all three mutations (Δ*speA*, *aguA:gm*, Δ*agu2ABCA’*) may be used to qualitatively “detect” exogenous agmatine as it is no longer capable of adding agmatine into a system, or removing it. To test this hypothesis we exposed this mutant to titrations of agmatine and similar molecules to determine if the response was specific to agmatine, and titratable. In Figure 4 the luminescence readout at 3 h confirms that the triple mutant with the *aguRB:lux* reporter is indeed capable of detecting agmatine in a dose dependent fashion. Though the dynamic range of this promotion system appears to range from 1 mM to 100 nM the effect is not linear relative to agmatine concentration. This may indicate that the promotion system becomes progressively less sensitive as the concentration increases to the top of the dynamic range potentially indicating saturation of the aguR protein or saturation of the reporter’s light production capabilities. Its vector control, which is genetically identical except missing the 1006 bp fragment containing the *aguRB* coding region*,* is not capable of responding to any of these molecules. It is apparent that there is a baseline residual expression of the *aguBA* operon as arginine and glutamine demonstrate a higher baseline in the reporter strain than the vector control. This data also suggests exogenously supplied putrescine may inhibit this reporter in high concentrations.

### 3.4. Use of the Agmatine Biosensor in Biologic Assays

To be useful as a “biosensor” the *P. aeruginosa* mutant needs to be able to report the presence of agmatine in a number of matrices. As *P. aeruginosa* is one of the most metabolically versatile microorganisms studied to date, there are a number of potential useful applications in the biologic sciences. The particular strain of *P. aeruginosa* used for this study is PA14, which has been shown to be “universally” pathogenic to organisms in multiple kingdoms including plants, animal, fungi, and insects and nematodes [16,23]. *P. aeruginosa* prefers aqueous environments, and as an “environmental” bacterium it can survive extremes of temperature and nutrient limitations. There are a number of theoretical advantages to using a “live” biosensor over analytical chemistry methods. There is minimal cost and time involved with preparing a culture of this organism and waiting for it to produce photons. There are a number of ways to detect light using plate based assays or gel documentation systems designed to detect luminescence, a very prevalent technology used to document western blots. The matrix may not require extensive processing, and in some cases, *P. aeruginosa* may invade and “find” the agmatine within a tissue source as it can invade the cell walls of diverse species. While the light output is reproducibly dose dependent, it is not likely to be quantitatively accurate enough to generate a standard curve, as too many biologic variables exist between agmatine binding AguR and light production. The biosensor also suffer some other technical limitations such as tissue depth, or matrix disruption of light transmittance, some of these can be overcome with more sensitive light detectors, or preparation of the matrix to remove layers of light blocking tissue. The *lux* operon also requires oxygen to fully function, so this sensor is not likely to be as useful in anaerobic environments. We also determined the luminescence to be significantly lower as temperatures drop below our assay temperature of 37 °C. In many instances the matrix can be “warmed” to 37 °C for measurement purposes alone as it is clear the *aguBA* operon is still active at 25 °C as was shown in our prior work [12]. We present three examples of our work with the agmatine biosensor below. We have also successfully utilized the biosensor in a number of other matrices including lettuce leaves, mouse lungs, and human sputum and urine (data not shown).

#### 3.4.1. Rapid Detection of Agmatine Secretion by Clinical *P. aeruginosa* Isolates

**Figure 5:**
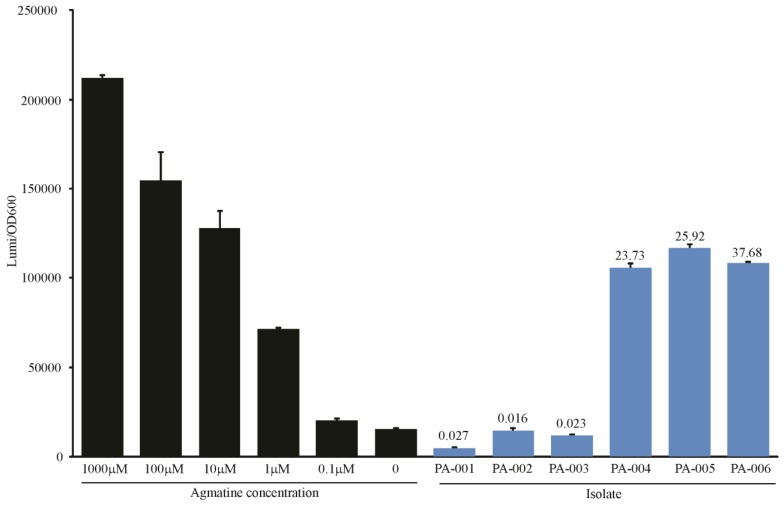
Detection of agmatine secretion in clinical isolates. The agmatine biosensor was grown in LB media with the indicated amount of agmatine, or grown in 50% LB and 50% spent supernatant from a 24 h liquid culture of clinical isolates. The y-axis represents the relative luminescence/OD_600_ of each well after 3 h growth. Values above the bars representing spent supernatants are agmatine concentrations of those supernatants as determined by UPLC-MS/MS in micromolar. Error bars are SEM of three wells.

The fate of agmatine within a bacterial system is dependent on how heavily the organism relies on the ornithine decarboxylase pathway *versus* the agmatine deiminase or agmatinase pathways to produce putrescine. We have studied the ability of a number of clinical isolates obtained from the sputum of patients with cystic fibrosis to either consume or create agmatine. In this work we discovered three mutants that hypersecrete agmatine (unpublished results). Figure 5 compares the biosensor readout compared to measurement by mass spectrometry to demonstrate the utility of the biosensor to identify these mutants. The cost of measuring 96 samples (via a plate assay) in the biosensor assay is the cost of the plate (~$6 USD) and minimal costs of media after purchase of the luminescence plate reader. The cost of processing and measuring 96 samples by mass spectrometry is over $1200 USD. The labor involved in preparing samples for injection into the mass spectrometer consumes at least 12 h of preparatory time. The biosensor assay requires about one hour to load the plate with samples, and results are revealed after a three-hour incubation. While mass spectrometry clearly offers more quantitative accuracy, the biosensor serves as a much more efficient and cost effective tool for screening purposes where dichotomous data can be distinguished.

#### 3.4.2. Detection of Arginine Decarboxylase Enzyme Activity

**Figure 6:**
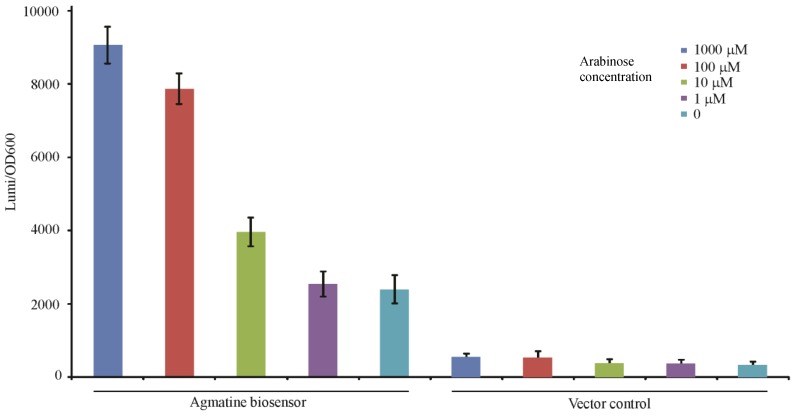
Monitoring arginine decarboxylase production through agmatine detection. The gene for arginine decarboxylase in *P. aeruginosa* (*speA*) was cloned without a promoter into the pBAD plasmid which uses a titratable arabinose promoter to induce cloned gene products. Each well contained 1 × 10^6^ CFU of the SpeA expressing *E. coli* strain and the agmatine biosensor or its vector control as described for Figure 3. The x-axis indicates which variant of the reporter strain is present and the concentration of arabinose in each culture starting at time 0. Each well also contained 1 mM arginine as a substrate for the reaction. The y-axis represents the relative luminescence/OD_600_ of the well at 10 h of growth. Error bars are SEM of three wells.

A co-culture experiment of the agmatine biosensor in *P.aeruginosa* and the *E. coli* strain harboring the arabinose induced *speA* gene was performed to assess the function of arginine decarboxylase relative to the concentration of arabinose in the context of 1 mM supplemental arginine as shown in Figure 6. The co-culture was induced with a logarithmic titration of arabinose from 1 mM to 1 µM. The concentration of agmatine detected by the biosensor increased relative to the concentration of arabinose. This indicates that the arabinose promotion system of pBad plasmid is titratable, like the *aguRB* agmatine response element as more arabinose results in more agmatine production.

#### 3.4.3. *In Vivo* Detection of Agmatine during Infection in Mouse Spinal Cords

Bioluminescence has been used to quantify gene transcription in multiple types of complex living systems including mammals [24]. The use of these *in vivo* imaging techniques to quantify bacterial gene expression while in an animal has also been successfully employed both with fluorescent and luminescent reporters [25,26]. *P. aeruginosa* can exist as a pathogen of multiple sites within the mammalian body, especially those that are immunocompromised [27]. Our use of the agmatine biosensor *in vitro* suggested that development of luminescence after agmatine exposure started to occur within the hour, but by 3 h demonstrated maximal differentiation between doses of agmatine.

**Figure 7:**
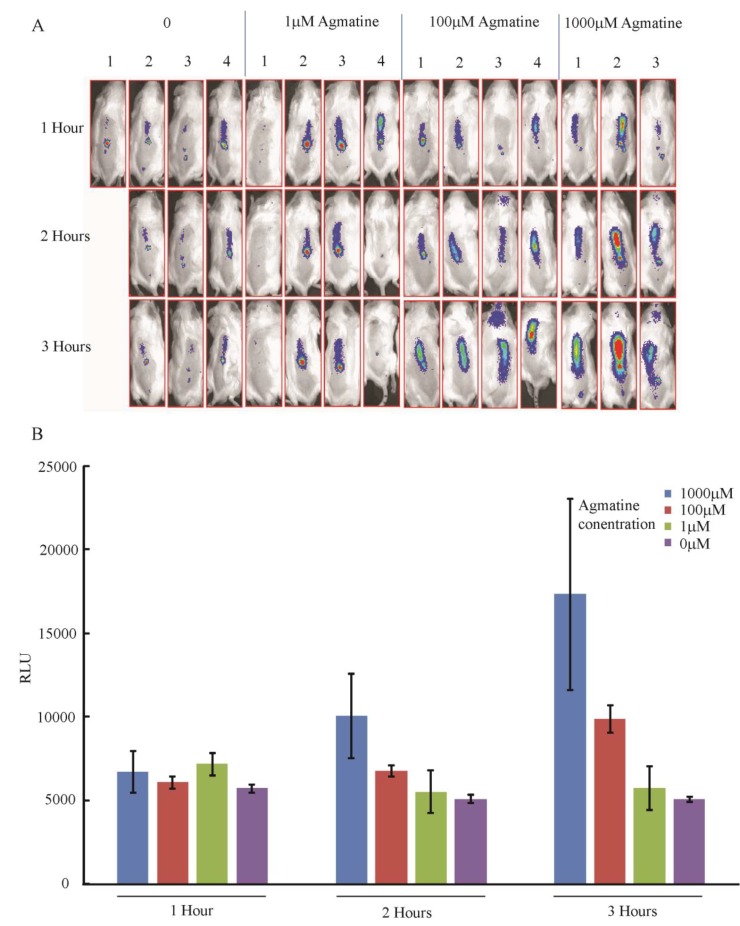
Detection of agmatine in mouse spinal cords. The agmatine biosensor *P. aeruginosa* was seeded with agmatine into the spinal columns of anesthetized mice. A Xenogen© *In Vivo* Imaging System was used to capture the luminescence produced in the alive but anesthetized mice every hour for three hours. Four mice per group were injected, missing images represent mice that died before imaging. (**A**) Images captured demonstrating intensity of luminescence over spinal cords; (**B**) Graphical representation of the average relative luminescence in each group of mice. Error bars indicate SEM.

Agmatine is being studied as a therapeutic agent in a number of clinical scenarios, especially as a neuromodulatory agent for pain control [4]. To determine if the agmatine biosensor could detect exogenously administered agmatine to the central nervous system we infected the spinal cords of mice with the agmatine biosensor. Mice were also co-administered agmatine over a range from 0 to 10 mM. Figure 7A,B demonstrate detection of luminescence in the spinal columns of live mice over 3 h of monitoring. There is a dose dependent titration of luminescence with increasing agmatine doses observed over time suggesting the biosensor is detecting agmatine *in vivo*.

## 4. Conclusions

Agmatine is a relatively understudied molecule with a wide range of potential biologic effects outside of its role as an intermediary to polyamine production. There is a growing body of literature to suggest a number of therapeutic applications could be made from agmatine or understanding pathways it affects. There are no commercially available assays to measure agmatine, and those analytic techniques to measure and detect agmatine are cumbersome and expensive. The development of a biosensor for agmatine in *Pseudomonas* offers a rapid, and inexpensive way to qualitatively detect agmatine. The biologic tool offers superior discrimination to related compounds, and allows for *in vivo* monitoring of agmatine in models capable of supporting growth of *Pseudomonas*. The biosensor could also be adapted to include other enzymatic or fluorescent gene products further expanding its sensitivity and application.
